# Stories of the future: manipulating RNA and Intra/Interkingdom communication

**DOI:** 10.1111/1751-7915.13344

**Published:** 2018-11-28

**Authors:** Cecilia Maria Arraiano

**Affiliations:** ^1^ Instituto de Tecnologia Química e Biológica António Xavier Universidade Nova de Lisboa Av. da República 2780‐157 Oeiras Portugal

My expertise is RNA and ribonucleases, and it has been a pleasure to observe and participate in the RNA revolution, which is rapidly changing how we interpret gene expression. Small non‐coding RNAs repress or activate genes in consonance to environmental or developmental cues and they can modulate life and death of the cell. RNAs can change shapes to adapt to different temperatures (thermosensors), and this can modify their functions; for instance, changing to 37°C can lead to structural changes that trigger the translation of virulence factors (Johansson *et al*., 2002). Furthermore, RNAs can sequester specific molecules to halt the production of surplus levels of those products (e.g. riboswitches – McCown *et al*., 2017). The advance of modern technologies such as RNAseq has shown that myriad RNAs are synthesized, and elegant methods can select specific types of RNAs and their targets (RNAs, proteins or DNA). About ten years ago, Nobel Prizes were given for RNA interference studies, the structure of the ribosome and the study of RNA polymerase II during transcription. Lessons from bacteria have led to the discovery of new DNA remodelling and immunological mechanisms mediated by RNA, CRISPR and Cas proteins. Many important genetic technologies have derived from this process and this will certainly lead to another Nobel in the near future.

The powerful RNAs have to exist in a balance between synthesis and degradation. Ribonucleases (RNases) actively process, degrade and monitor the quality control of all types of RNA (Arraiano *et al*., 2010, 2013; Saramago *et al*., 2014). They can recognize the sequence, structure and the terminal tails of some of their favourite targets. This process is very dynamic, and the ‘predator’ RNases have to locate their ‘prey’ RNAs in time and space. The ribonucleases have a fundamental role in the turnover of RNAs since the accumulation of degradation products and aberrant transcripts is usually quite detrimental for the cell; furthermore, they actively participate in the recycling of valuable nucleotides. Engineering and modulating the lifespan of RNAs has great potential (Viegas *et al*., 2018) and will certainly be one of the highlights of future science. Synthetic Biology supported by all the recent RNA‐derived technologies will lead to important Biotech applications. (de Lorenzo, 2018).

The investigation of new small non‐coding RNAs in a symbiont bacteria gave me the inspiration for this article within the frame of the Microbial Biotechnology Crystal‐Ball series of 2018/2019. The bacterium was the betaproteobacteria *Herbaspirillum seropedicae* strain SmR1, an endophyte that colonizes economically important grain crops including rice, maize and fixes nitrogen, thereby promoting plant growth (Pedrosa *et al*., 2001). During the laboratory experiments, it struck me that when naringenin (a plant flavonoid) is added *H. seropedicaea* acts as if it was inside the host plant (Tadra‐Sfeir *et al*., 2015). This important symbiont senses it is somewhere else and behaves in conformity with the ‘fake’ environment, just like ‘virtual reality’. The exploration of this concept can have a tremendous potential for innumerous applications, and the ‘make believe’ between interkingdom communication is my Crystal Ball proposition for a future story of success!

It is already known that bacteria can easily communicate by means such as quorum sensing, and the molecular messengers containing the information sometimes are only ‘translated’ by certain populations (Bassler and Losick, 2006). Besides, some bacteria can intercept and hijack the information sent by other species (Xavier and Bassler, 2005). The small non‐coding RNAs are among the molecules participating in this dynamic network of communication. The studies on the microbiome will progress much faster when we learn to communicate with bacteria.

Recent developments have shown that the interactions of bacteria among each other and with different organisms can also be mediated by volatile molecules (Audrain *et al*., 2015; Chung *et al*., 2016). It is certainly a powerful means, especially if bacteria want to connect to distant targets. In other species, it is already known that scent molecules can repel or attract certain organisms. Modern technologies have been developed to be able to detect, analyse and then manufacture these new molecules. This will be extremely useful to establish a rapid interkingdom wide web of communication.

The production of extracellular vesicles (EVs) is a universal feature of eukaryotes as well as prokaryotes. These extracellular vesicles have also been referred to as exosomes and microvesicles. They are small vesicles with phospholipid membrane carrying proteins DNA and RNAs and can serve as mediators of information in intercellular communication, inter‐ and intraspecies communication and molecular mimicry. The vesicles make selective sorting of cargo, but all major RNA classes can be present and delivered to bacterial and/or eukaryotic cells. Therefore, RNA carried by these EVs can be considered as a universal mediator of communication (Tsatsaronis *et al*., 2018).

The gastrointestinal tract is a place where communication constantly occurs. Membrane vesicles transmit signals and facilitate group activities and there is much interkingdom communication. For instance, miRNA‐loaded EVs secreted by nematodes are internalized by host mice cells and suppress host immune response. Certain EVs have also been linked to gastrointestinal cancer (Barteneva *et al*., 2017). Small RNAs from plants, bacteria and fungi are ubiquitous in human plasma. Bacterial RNAs are a stimulus for innate immune responses and it has been shown that specific interference can be triggered by ingested double‐stranded RNA (Nobel Prize 2006 – Craig and Mello). EVs derived from host eukaryotic cells meet in the intraluminal space and interact with the vesicles from prokaryotic symbiotic cells, edible plants, fungi and parasites. If we consider that the intestine is colonized by approximately 100 trillion microorganisms comprising hundreds of different species, it is obvious that the intestinal EVs are a huge reservoir of microbial and parasitic antigens. Therefore, the role of EVs is an exciting frontier with important implications in the dynamics of host–pathogen interactions and vaccine applications. The possibility of applying genome editing tools to engineer EVs loaded with ‘tailored’ cargoes is an exciting future perspective to transmit the desired information to other species.

It has been said that the microbiome can act in a gut‐brain connection but we can certainly learn a lot from other examples, some of which still look like a scary episode of science fiction. Certain fungi can infect ants and completely change their behaviour. These ants are prone to move to locations favourable to the development of the fungi, and move like robots: the ‘zombie ants’ (Hughes *et al*., 2011). The scariest and probably most interesting fact to be investigated is that it was shown that the brain of the ant is unaltered, so as the ant is able to move properly to the location chosen by the fungi (Fredericksen *et al*., 2017). Other examples have been also described. For instance, *Toxoplasma gondii* infects mice and rats and makes them lose fear of cats (Berdoy *et al*., 2000). The parasite re‐engineers the rat′s brain and the behavioural change makes them an easy prey for cats. This change in behaviour induced by the interspecies communication leads to a fast catch by the cats – which are the preferred host of *Toxoplasma gondii*. And the ‘fatal attraction’ persists even after the infection is cleared (Ingram *et al*., 2013).

RNA is ubiquitous, susceptible to modifications, and has high versatility in structure, sequence and size. Besides, RNA can have a very fast turnover. All these features make RNA modules easy vehicles to sense and communicate in a global internet species communication.

Small nucleotides can be slightly modified and be like a wire to connect and alter information (Nelson and Breaker, 2017). For instance, c‐di‐GMP (cyclic di‐guanosine‐monophosphate) is a second messenger used in signal transduction in a wide variety of bacteria. It has shown to be quite important for biofilm formation, can work as an allosteric activator of enzymes, has implications for pathogenicity, and can be linked to RNases and riboswitches, among others. Another example is the global regulatory nucleotide ppGpp (guanosine tetraphosphate‐the magic spot). The ppGpp is an alarmone which can be crucial to re‐direct RNA polymerase to certain promoters, which is important under certain stresses, namely stringent response.

The evidence has been increasing that RNA modifications (in the so‐called epitranscriptome) are far more relevant than previously suspected (Nachtergaele and He, 2017). RNA modifications are present in the three kingdoms of life. Advances in technology are showing that these ‘pontuations and synthax’ in the RNA alphabet can give new meanings to apparently similar messages.

In conclusion, our understanding of intra and interkingdom communication is still in its infancy but brings great promises. It can be used to produce a new form of antibiotics that restrict and tame pathogens and can also be used to ‘motivate’ microbes to produce many powerful and useful molecules, some of which have yet to be discovered. Therefore, my Crystal Ball is that RNA and Intra/Interkingdom communication will drive the most exciting progress in microbiology over the next few years.
